# Supplementary material for: Association between obesity and risk of fracture, bone mineral density and bone quality in adults: A systematic review and meta-analysis
Source: PLoS One. 2021 Jun 8;16(6):e0252487. doi: 10.1371/journal.pone.0252487 (PMC8186797; doi:10.1371/journal.pone.0252487)
Supplement: S1 Table — (DOCX) [file pone.0252487.s002.docx]

**S1 Table. Search strategy.**

| **Database** | **#** | **Concept** | **Search strategy** |
| --- | --- | --- | --- |
|  |  |  |  |
| PubMed | #1 | Exposition = Obesity | "Obesity"[MeSH] OR obesity[TIAB] OR obese[TIAB] OR “excess fat”[TIAB] OR “excess body fat”[TIAB] OR “excess weight”[TIAB] OR “excessive weight”[TIAB] OR “excess body weight”[TIAB] |
|  | #2 | Outcome = Bone mineral density | bone mineral density[TIAB] OR bone density[TIAB] OR bone mass[TIAB] OR “Bone Density”[MeSH] OR "Bone and Bones"[Mesh:NoExp] |
|  | #3 | Outcome = Bone quality | bone quality[TIAB] OR bone microarchitecture[TIAB] OR Bone characteristics[TIAB] OR Bone structure[TIAB] OR bone turnover*[TIAB] OR bone metabolism[TIAB] OR bone remodeling[TIAB] OR bone resorption[TIAB] OR bone resorption[MeSH] OR bone formation[TIAB] OR osteogenesis[MeSH] OR "Bone and Bones"[Mesh:NoExp] OR CTX[TIAB] OR C-terminal telopeptide[TIAB] OR collagen type I trimeric cross-linked peptide[MeSH] OR N-terminal telopeptide[TIAB] OR NTX[TIAB] OR osteocalcin[TIAB] OR osteocalcin[MeSH] OR sclerostin[TIAB] OR P1NP[TIAB] OR procollagen type 1 N-terminal peptide[MeSH] OR “bone alkaline phosphatase”[TIAB] |
|  | #4 | Outcome = fracture | fracture*[TIAB] OR “Fractures, Bone”[MeSH] OR “broken bone”[TIAB] |
|  | #5 | Study design | transversal[TIAB] OR cross-sectional[TIAB] OR Cross-Sectional Studies[MeSH]  OR  cohort*[TIAB] OR prospective[TIAB] OR cohort studies[MeSH] OR Longitudinal[TIAB] OR prospective[TIAB] OR longitudinal study[MeSH] OR prospective study[MeSH] OR follow-up[TIAB]  OR  clinical trial[TIAB] OR clinical trial[MeSh] OR epidemiologic methods[MeSH:NoExp] OR cohort analysis[MeSH] OR case-control study[MeSH] OR case-control stud*[TIAB]  OR  randomized controlled trial[PT] OR controlled clinical trial[PT] OR random*[TIAB] OR placebo*[TIAB] OR single blind*[TIAB] OR double blind*[TIAB] OR triple blind*[TIAB] OR Trial*[TIAB] OR Group*[TIAB] OR retrospectiv*[TIAB] OR interrupted time serie*[TIAB] OR experimental stud*[TIAB] OR quasi-experimental stud*[TIAB] |
|  | #6 | Filter | NOT (Animals[MeSH] NOT humans[MeSH]) |

| **Database** | **#** | **Concept** | **Search strategy** |
| --- | --- | --- | --- |
|  |  |  |  |
| EMBASE | #1 | Exposition = Obesity | ‘Obesity’:exp OR ‘obesity’:ti,ab,kw OR ‘obese’:ti,ab,kw OR ‘excess fat’:ti,ab,kw OR ‘excess body fat’:ti,ab,kw OR ‘excess weight’:ti,ab,kw OR ‘excessive weight’:ti,ab,kw OR ‘excess body weight’:ti,ab,kw |
|  | #2 | Outcome = Bone mineral density | ‘bone mineral density’:ti,ab,kw  OR ‘bone density’:ti,ab,kw OR ‘bone mass’:ti,ab,kw OR ‘Bone’:de OR ‘bone density’:exp |
|  | #3 | Outcome = Bone quality | ‘bone quality’:ti,ab,kw OR ‘bone microarchitecture’:ti,ab,kw OR ‘bone microarchitecture’:exp OR ‘bone turnover*’:ti,ab,kw OR ‘bone metabolism’:ti,ab,kw OR ‘bone remodeling’:ti,ab,kw  OR ‘bone resorption’:ti,ab,kw OR ‘bone formation’:ti,ab,kw OR 'bone characteristics’:ti,ab,kw OR ‘bone structure’:ti,ab,kw OR 'bone characteristics and functions':exp OR ‘bone development’:exp OR ‘CTX’:ti,ab,kw OR ‘C-terminal telopeptide’:ti,ab,kw OR ‘NTX’:ti,ab,kw OR ‘N-terminal telopeptide’:ti,ab,kw OR ‘osteocalcin’:ti,ab,kw OR ‘osteocalcin’:exp OR ‘sclerostin’:exp OR ‘sclerostin’:ti,ab,kw OR ‘P1NP’:ti,ab,kw OR ‘bone alkaline phosphatase’:ti,ab,kw |
|  | #4 | Outcome = fracture | ‘fracture*’:ti,ab,kw OR ‘fracture*’:exp OR ‘broken bone*’:ti,ab,kw |
|  | #5 | Study design | ‘Transversal’:ti,ab,kw OR ‘cross-sectional’:ti,ab,kw OR ‘Cross-Sectional Studies’:de OR ‘cohort*’:ti,ab,kw OR ‘prospective’:ti,ab,kw OR ‘cohort analysis’:exp OR ‘prospective study’:exp OR ‘follow-up’:exp OR ‘longitudinal study’:ti,ab,kw OR ‘prospective study’:ti,ab,kw OR ‘clinical trial’:ti,ab,kw OR ‘clinical study’:exp OR ‘case-control study’:exp OR case-control stud*:ti,ab,kw OR ‘controlled clinical trial’:exp OR ‘randomized controlled trial’:exp OR ‘random*’:ti,ab,kw OR ‘placebo*’:ti,ab,kw OR ‘single blind*’:ti,ab,kw OR ‘double blind*’:ti,ab,kw OR ‘triple blind*’:ti,ab,kw OR ‘trial*’:ti,ab,kw OR ‘group*’:ti,ab,kw OR ‘retrospectiv*’:ti,ab,kw OR ‘interrupted time serie*’:ti,ab,kw OR ‘experimental stud*’:ti,ab,kw OR ‘quasi-experimental stud*’:ti,ab,kw |
|  | #6 | Filter | AND [embase]/lim |
|  | #7 | Filter | NOT (‘Animal’:de NOT ‘human’:de) |

| **Database** | **#** | **Concept** | **Search strategy** |
| --- | --- | --- | --- |
|  |  |  |  |
| Web of science | #1 | Exposition = Obesity | TS=Obesity OR TS=Obese OR TS=excess fat OR TS=excess body fat OR TS=excess weight OR TS=excessive weight OR TS=excess body weight |
|  | #2 | Outcome = Bone mineral density | TS=bone mineral density OR TS=bone density OR TS=Bone mass |
|  | #3 | Outcome = Bone quality | TS=bone quality OR TS=bone microarchitecture OR TS= Bone characteristics OR TS=Bone structure OR TS=bone turnover* OR TS=bone metabolism OR TS=bone remodeling OR TS=bone resorption OR TS=bone formation OR TS=Osteogenesis OR TS=Bone* OR TS=CTX OR TS=C-terminal telopeptide OR TS=NTX OR TS=N-terminal telopeptide OR TS=osteocalcin OR TS=P1NP OR TS=procollagen type 1 intact N-terminal propeptide OR TS=sclerostin OR TS=bone alkaline phosphatase |
|  | #4 | Outcome = fracture | TS=fracture* OR TS=broken bone* |
|  | #5 | Study design | TS=transversal OR TS=cross-sectional* OR TS=cohort* OR TS=prospective OR TS=longitudinal OR TS=clinical trial OR TS=randomized controlled trial OR TS=controlled clinical trial OR TS=random* OR TS=Trial* OR TS=Group* OR TS=placebo* OR TS=single blind* OR TS=double blind* OR TS=triple blind* OR TS=Follow-up OR TS=case-control stud* OR TS=retrospectiv* OR TS=interrupted time serie* OR TS=experimental stud* OR TS=quasi-experimental stud* |

| **Database** | **#** | **Concept** | **Search strategy** |
| --- | --- | --- | --- |
|  |  |  |  |
| Cochrane | #1 | Exposition = Obesity | [mh ”Obesity”] OR (obesity):ti,ab OR (obese):ti,ab OR (excess fat):ti,ab OR (excess body fat):ti,ab OR (excess weight):ti,ab OR (excessive weight):ti,ab OR (excess body weight):ti,ab |
|  | #2 | Outcome = Bone mineral density | (bone mineral density):ti,ab OR (bone density):ti,ab OR [mh ”Bone Density”] OR (bone mass):ti,ab OR [mh ^”Bone and Bones”] |
|  | #3 | Outcome = Bone quality | (bone quality):ti,ab OR (bone microarchitecture):ti,ab OR (bone turnover*):ti,ab OR (bone metabolism):ti,ab OR (bone remodeling):ti,ab  OR (bone resorption):ti,ab OR (bone formation):ti,ab OR (bone structure):ti,ab OR (bone characteristics):ti,ab OR [mh ^”Bone and Bones”] OR [mh ^”Bone development”] OR (CTX):ti,ab OR (C-terminal telopeptide):ti,ab OR (NTX):ti,ab OR (N-terminal telopeptide):ti,ab OR (osteocalcin):ti,ab OR [mh ”osteocalcin”] OR (sclerostin):ti,ab OR (P1NP):ti,ab OR (procollagen type 1 intact N-terminal propeptide):ti,ab OR (bone alkaline phosphatase):ti,ab |
|  | #4 | Outcome = fracture | (fracture*):ti,ab OR (broken bone*):ti,ab |
|  | #5 | Study design | (Transversal):ti,ab OR (cross-sectional):ti,ab OR (cohort*):ti,ab OR (prospective):ti,ab OR (clinical trial):ti,ab OR [mh ^“Cross-Sectional Studies”] OR [mh “clinical study”] OR [mh “controlled clinical trial”] OR (random*):ti,ab OR (Trial*):ti,ab OR (Group*):ti,ab OR (Longitudinal):ti,ab OR (Follow-up):ti,ab OR (placebo*):ti,ab OR (single blind*):ti,ab OR (double blind*):ti,ab OR (triple blind*):ti,ab OR (case-control stud*):ti,ab OR (retrospectiv*):ti,ab OR (interrupted time serie*):ti,ab OR (experimental stud*):ti,ab OR (quasi-experimental stud*):ti,ab |
