# Supplementary material for: Association between obesity and risk of fracture, bone mineral density and bone quality in adults: A systematic review and meta-analysis
Source: PLoS One. 2021 Jun 8;16(6):e0252487. doi: 10.1371/journal.pone.0252487 (PMC8186797; doi:10.1371/journal.pone.0252487)
Supplement: S2 Table — (DOCX) [file pone.0252487.s003.docx]

| **S2 Table. Study characteristics of included studies for bone turnover markers outcome.** | | | | | | | | |
| --- | --- | --- | --- | --- | --- | --- | --- | --- |
| Study | Country | Study design (sample size) | Sample size by group | Obesity criterion | Age (mean ± SD) | Sex (% female) | Bone turnover markers assessed | Quality score^a^ |
| **Postmenopausal women** | | | | | | | | |
| Bilic-Curcic 2017 | Croatia | CS (114) | OB: 83  NO: 31 | OB: BMI>27  NO: BMI≤27 | ≥45 | 100 | OCN, CTX | 5 |
| Caglar 2014 | Turkey | CS (87) | OB: 60  NO: 27 | OB: BMI≥30  NO: BMI<30 | 54.7 ± 5.3 | 100 | OCN | 5 |
| Cakmak 2005 | Turkey | Cohort (64) | OB: 38  NO: 26 | OB: BMI≥25  NO: BMI<25 | 56.5 ± 6.9 | 100 | OCN, CTX | 5 |
| Carsote 2019 | Romania | CS (56) | OB: 39  NO: 17 | OB: BMI≥25  NO: BMI 18.5-24.9 | OB: 47.8 ± 9.4  NO: 47.5 ± 5.0 | 100 | P1NP, OCN, CTX | 6 |
| Cifuentes 2003 | USA | CS (210) | OB: 124  NO: 86 | OB: BMI≥25  NO: BMI<25 | 67 ± 6 | 100 | OCN | 7 |
| Garcia-Martin 2011 | Spain | CS (54) | OB: 23  NO: 31 | OB: BMI≥30  NO: BMI<30 | NR | 100 | OCN | 8 |
| Genazzani 2001 | Italy | Cohort (31) | OB: 15  NO: 16 | OB: BMI≥25  NO: BMI<25 | Range 50-65 | 100 | OCN | 6 |
| Holecki 2007 | Poland | Case-control (62) | OB: 43  NO: 19 | NR | OB: 50.1 ± 4.5  NO: 53.8 ± 5.2 | 100 | OCN, CTX | 6 |
| Ibrahim 2011 | Egypt | CS (74) | OB: 37  NO: 37 | OB: BMI>30  NO: BMI<25 | OB: 57.4 ± 4.4  NO: 56.6 ± 3.5 | 100 | OCN | 7 |
| Jiajue 2014 | China | CS (1,410) | OB: 810  NO: 600 | OB: BMI≥25  NO: BMI<25 | OB: 64.0 ± 15.3  NO: 65.6 ± 15.9 | 100 | P1NP, CTX | 5 |
| Kim 2016 | Korea | CS (124) | OB: 52  NO: 72 | OB: BMI≥25  NO: BMI<25 | OB: 60.2 ± 6.7  NO: 59.6 ± 7.4 | 100 | OCN, CTX | 8 |
| Kyvernitakis 2014 | Germany | Cohort (70) | OB: 44  NO: 26 | OB: BMI>25  NO: BMI 18-25 | 62.7 ± 7.5 | 100 | P1NP, CTX | 6 |
| Lee 2012 | Korea | CS (214) | OB: 152  NO: 62 | OB: BMI≥25  NO: BMI<25 | 55.7 ± 5.1 | 100 | OCN | 8 |
| Machado 2016 | Brazil | Cohort (433) | OB: 266  NO: 167 | OB: BMI>27  NO: BMI<27 | OB: 72.7 ± 5.7  NO: 74.9 ± 8.1 | 100 | P1NP, CTX | 7 |
| Olmos 2018 | Spain | Cohort (2,597) | OB: 2094  NO: 503 | OB: BMI≥25  NO: BMI<25 | OB: 65.4 ± 13.4  NO: 61.0 ± 10.2 | 70.3 | P1NP, CTX | 6 |
| Ostrowska 2011 | Poland | CS (47) | OB: 29  NO: 18 | NR | OB: 57.7 ± 8.2  NO: 59.5 ± 3.5 | 100 | OCN | 6 |
| Ribot 1987 | France | CS (176) | OB: 77  NO: 99 | NR | OB: 53.2 ± 6.0  NO: 53.1 ± 5.7 | 100 | OCN | 1 |
| Shaarawy 2003 | Egypt | CS (90) | OB: 37  NO: 53 | OB: BMI>30  NO: BMI 20-25 | 58.8 ± 0.5 | 100 | OCN, NTX | 4 |
| Sodi 2009 | UK | CS (71) | OB: 40  NO: 31 | OB: BMI≥25  NO: BMI<25 | OB: 68.7  NO: 68.6 | 100 | P1NP, CTX | 7 |
| Sornay-Rendu 2013 | France | Case-control (189) | OB: 63  NO: 126 | OB: BMI≥30  NO: BMI 18.5-24.9 | OB: 68.6 ± 7  NO: 68.2 ± 7.4 | 100 | P1NP, OCN, CTX | 8 |
| Tanaka 2013 | Japan | Cohort (1,479) | OB: 348  NO: 1131 | OB: BMI≥25  NO: BMI 18.5-24.9 | OB: 63.2 ± 10.1  NO: 62.5 ± 11.2 | 100 | OCN, NTX | 5 |
| Tay 2018 | Singapore | Cohort (30) | OB: 10  NO: 20 | OB: BMI≥30  NO: BMI<30 | OB: 65.3 ± 9.3  NO: 61.7 ± 13.4 | 70 | P1NP, CTX | 7 |
| Zhou 2010 | China | CS (1,479) | OB: 750  NO: 729 | OB: BMI≥25  NO: BMI<25 | OB: 57.5 ± 7.4  NO: 56.8 ± 5.8 | 100 | OCN, NTX | 5 |
| **Premenopausal women** | | | | | | | | |
| Lingaiah 2019 | Finland | Cohort (61) | OB: 27  NO: 34 | OB: BMI≥27  NO: BMI<27 | OB: 27.3 ± 5.0  NO: 27.9 ± 4.2 | 100 | P1NP, CTX | 7 |
| Pereira 2007 | Brazil | CS (39) | OB: 28  NO: 11 | OB: BMI≥30  NO: BMI<30 | OB: 37.8 ± 1.7  NO: 37.2 ± 3.1 | 100 | OCN | 6 |
| Sowers 2013 | USA | Cohort (908) | OB: 490  NO: 418 | OB: BMI≥25  NO: BMI<25 | OB: 46.0 ± 3.8  NO: 45.8 ± 3.8 | 100 | NTX | 6 |
| Yaylali 2019 | Turkey | CS (128) | OB: 73  NO: 55 | OB: BMI≥30  NO: BMI<30 | NR | 100 | OCN | 6 |
| **Men** | | | | | | | | |
| Gu 2017 | China | CS (70) | OB: 44  NO: 26 | OB: BMI≥25  NO: BMI 18-24.9 | OB: 53.0 ± 8.6  NO: 51.5 ± 6.1 | 0 | OCN | 6 |
| Kanazawa 2008 | Japan | CS (163) | OB: 73  NO: 90 | OB: BMI≥24  NO: BMI<24 | OB: 56.8 ± 21.0  NO: 58.6 ± 15.3 | 0 | OCN, NTX | 7 |
| Tencerova 2019 | Denmark | CS (54) | OB: 35  NO: 19 | OB: BMI≥25  NO: BMI<25 | OB: 34.8 ± 2.6  NO: 31.0 ± 3.0 | 0 | P1NP, CTX | 7 |
| **Mixed population** | | | | | | | | |
| Albassam 2019 | Saudi Arabia | CS (265) | OB: 179  NO: 86 | OB: BMI≥30  NO: BMI<30 | OB: 50.6 ± 7.5  NO: 52.7 ± 8.1 | 100 | OCN, Sclerostin | 7 |
| Azzam 2019 | Egypt | CS (75) | OB: 54  NO: 21 | OB: BMI≥25  NO: BMI 18.5-24.9 | OB: 33.9 ± 8.9  NO: 28.7 ± 2.9 | 68 | Sclerostin | 6 |
| De Araujo 2017 | Brazil | Case-control (78) | OB: 54  NO: 24 | NR | OB: 53.0 ± 13.6  NO: 55 ± 7 | 57.7 | OCN, CTX | 3 |
| El-Eshmawy 2015 | Egypt | Case-control (150) | OB: 100  NO: 50 | OB: BMI≥25  NO: BMI<25 | OB: 45.9 ± 5.0  NO: 44.8 ± 5.7 | 54 | OCN | 7 |
| Evans 2015 | UK | CS (223) | OB: 146  NO: 77 | OB: BMI≥30  NO: BMI 18.5-24.9 | OB: 49.8 ± 9.9  NO: 49.8 ± 9.8 | 50.7 | P1NP, CTX | 8 |
| Grethen 2012 | USA | Case-control (40) | OB: 20  NO: 20 | NR | OB: 44.4 ± 8.9  NO: 41.3 ± 6.9 | 100 | NTX, Sclerostin | 7 |
| Jacobs 2018 | USA | Epidemiological (600) | OB: 318  NO: 282 | OB: BMI≥30  NO: BMI<30 | OB: 59.6 ± 11.8  NO: 63.1 ± 9.1 | 58.2 | CTX | 7 |
| Kadric 2018 | Germany | CS (3,583) | OB: 2483  NO: 1100 | OB: BMI≥25  NO: BMI<25 | NR | 50.3 | CTX | 7 |
| Lim 2013 | Korea | Cohort (25) | OB: 16  NO: 9 | OB: BMI>25  NO: BMI≤25 | OB: 23.3 ± 0.2  NO: 24.6 ± 0.3 | 52 | OCN, CTX | 8 |
| Pham 2020 | Finland | CS (32) | OB: 23  NO: 9 | NR | OB: 47.0 ± 1.9  NO: 46.8 ± 2.0 | 81.3 | CTX | 6 |
| Saarnio 2018 | Finland | CS (548) | OB: 306  NO: 242 | OB: BMI≥25  NO: BMI 18.5-24.9 | OB: 42.2 ± 5.8  NO: 42.0 ± 4.1 | 64.5 | P1NP, OCN, CTX | 7 |
| Sukumar 2011 | USA | Case-control (111) | OB: 52  NO: 59 | OB: BMI>35  NO: BMI<27 | OB: 52.7 ± 11.7  NO: 50.6 ± 8.5 | 100 | P1NP, OCN, NTX | 8 |
| Tonks 2017 | Australia | CS (69) | OB: 51  NO: 18 | OB: BMI>25  NO: BMI≤25 | OB: 58.8 ± 15.0  NO: 53.8 ± 8.1 | 50.7 | P1NP, OCN, CTX | 8 |
| Viljakainen 2014 | Sweden | Case-control (68) | OB: 34  NO: 34 | NR | OB: 19.3 ± 2.3  NO: 19.4 ± 2.3 | 58.8 | P1NP, OCN, CTX | 8 |
| Zhao 2020 | China | Case-control (79) | OB: 50  NO: 29 | OB: BMI>25  NO: BMI≤25 | OB: 53.6 ± 10.4  NO: 59.0 ± 8.6 | 57.0 | OCN | 5 |
| **Studies not included in the meta-analysis** | | | | | | | | |
| Barghash 2014 | Saudi Arabia | Case-control (120) | OB: 80  NO: 40 | OB: BMI≥25  NO: BMI<25 | 41.5 (median) | 0 | OCN | 5 |
| Xu 2018 | China | CS (1,179) | OB: 327  NO: 852 | OB: BMI≥25  NO: BMI<25 | OB: 58.9 ± 6.3  NO: 59.8 ± 6.2 | 60.6 | OCN | 6 |
| Wyskida 2020 | Poland | CS (98) | OB: 78  NO: 20 | OB: BMI≥25  NO: BMI<25 | OB: 27.8 ± 5.7  NO: 24.0 ± 4.0 | 100 | Sclerostin | 6 |

CS: cross-sectional; OB: obese; NO: non-obese; BMI: Body Mass Index; WC: Waist circumference; OCN: osteocalcin, P1NP: Procollagen type 1 intact N-terminal propeptide; CTX: C-terminal telopeptide; NTX: N-terminal telopeptide.

BMI is expressed in kg/m^2^.

WC is expressed in cm.

^a^Quality score was obtained from the Joanna Briggs Institute tool (JBI): <4: high risk of bias; 4-6 moderate risk of bias; ≥7 low risk of bias.
