# Supplementary material for: Association between obesity and risk of fracture, bone mineral density and bone quality in adults: A systematic review and meta-analysis
Source: PLoS One. 2021 Jun 8;16(6):e0252487. doi: 10.1371/journal.pone.0252487 (PMC8186797; doi:10.1371/journal.pone.0252487)
Supplement: S3 Table — (DOCX) [file pone.0252487.s004.docx]

| **S3 Table. Assessment methods used for bone turnover markers.** | | | | | |
| --- | --- | --- | --- | --- | --- |
| Study | Bone turnover markers assessed | Time of day | Fasting? | Specimen type | Assay used |
| Bilic-Curcic 2017 | OCN, CTX | NR | NR | OCN: Serum  CTX: Urine | OCN: IRMA  CTX: ECLIA |
| Caglar 2014 | OCN | Morning | Yes | Serum | ECLIA |
| Cakmak 2005 | OCN, CTX | Morning | Yes | Serum | OCN: ELISA  CTX: ECLIA |
| Carsote 2019 | P1NP, OCN, CTX | NR | NR | NR | P1NP: ECLIA  OCN: ECLIA  CTX: ECLIA |
| Cifuentes 2003 | OCN | Morning | Yes | Serum | RIA |
| Garcia-Martin 2011 | OCN | Morning | Yes | Serum | ECLIA |
| Genazzani 2001 | OCN | NR | NR | Plasma | ELISA |
| Holecki 2007 | OCN, CTX | Morning | Yes | Serum | ECLIA |
| Ibrahim 2011 | OCN | Morning | Yes | Serum | ELISA |
| Jiajue 2014 | P1NP, CTX | Morning | Yes | Serum | ECLIA |
| Kim 2016 | OCN, CTX | NR | NR | Serum | OCN: Y-Counter  CTX: Molecular Analytics |
| Kyvernitakis 2014 | P1NP, CTX | Morning | Yes | Serum | P1NP: ECLIA  CTX: ECLIA |
| Lee 2012 | OCN | Morning | Yes | Serum | ELISA |
| Machado 2016 | P1NP, CTX | NR | NR | Serum | ECLIA |
| Olmos 2018 | P1NP, CTX | Morning | Yes | Serum | ECLIA |
| Ostrowska 2011 | OCN | Morning | NR | Serum | ELISA |
| Pham 2020 | CTX | NR | NR | Serum | CTX: ELISA |
| Ribot 1987 | OCN | NR | NR | Serum | IRMA |
| Shaarawy 2003 | OCN, NTX | Morning | Yes | OCN: Serum  NTX: Urine | OCN: IRMA  NTX: ELISA |
| Sodi 2009 | P1NP, CTX | NR | NR | NR | P1NP: ECLIA  CTX: ECLIA |
| Sornay-Rendu 2013 | P1NP, OCN, CTX | Morning | Yes | Serum | ECLIA |
| Tanaka 2013 | OCN, NTX | NR | NR | NR | NR |
| Tay 2018 | P1NP, CTX | NR | Yes | NR | P1NP: IRMA  CTX: ELISA |
| Tencerova | P1NP, CTX | Morning | Yes | Serum | P1NP: Immunoassay  CTX: Immunoassay |
| Yaylali 2019 | OCN | Morning | Yes | Serum | ECLIA |
| Zhou 2010 | OCN, NTX | Morning | NR | OCN: Plasma  NTX: Urine | OCN: IRMS  NTX: ELISA |
| Lingaiah 2019 | P1NP, CTX | NR | Yes | Serum | ECLIA |
| Pereira 2007 | OCN | Morning | Yes | Serum | IRMA |
| Sowers 2013 | NTX | NR | NR | Urine | ELISA |
| Gu 2017 | OCN | Morning | NR | NR | ELISA |
| Kanazawa 2008 | OCN, NTX | Morning | Yes | OCN: Serum  NTX: Urine | OCN: RIA  NTX: ELISA |
| Albassam 2019 | OCN, Sclerostin | Morning | Yes | Serum | Multiplex assay |
| Azzam 2019 | Sclerostin | NR | Yes | Serum | ELISA |
| De Araujo 2017 | OCN, CTX | Morning | Yes | Serum | OCN: EASIA  CTX: ECLIA |
| El-Eshmawy 2015 | OCN | Morning | Yes | Plasma | ECLIA |
| Evans 2015 | P1NP, CTX | Morning | Yes | Serum | ECLIA |
| Grethen 2012 | NTX, Sclerostin | NR | NR | Serum | ELISA |
| Jacobs 2018 | CTX | NR | NR | Urine | ELISA |
| Kadric 2018 | CTX | Morning and noon | Yes (61%) | Serum | ECLIA |
| Lim 2013 | OCN, CTX | Morning | Yes | Serum | RIA |
| Saarnio 2018 | P1NP, OCN, CTX | Morning | Yes | Serum | P1NP + CTX: ECLIA  OCN: Two-site immunoassay |
| Sukumar 2011 | P1NP, OCN, NTX | NR | NR | Serum | NTX : ELISA  OCN + P1NP : RIA |
| Tonks 2017 | P1NP, OCN, CTX | NR | Yes | Serum | ECLIA |
| Viljakainen 2014 | P1NP, OCN, CTX | NR | NR | Serum | P1NP + CTX: ECLIA  OCN: Two-site immunoassay |
| Barghash 2014 | OCN | Morning | Yes | Plasma | ELISA |
| Wyskida 2020 | Sclerostin | Morning | Yes | Plasma | ELISA |
| Xu 2018 | OCN | Morning | Yes | Serum | ECLIA |
| Zhao 2020 | OCN | Morning | Yes | NR | NR |

NR: Not reported; OCN: Osteocalcin; P1NP: Procollagen type 1 intact N-terminal propeptide; CTX: C-terminal telopeptide; NTX: N-terminal telopeptide; IRMA: Immunoradiometric assay; ECLIA: Electrochemiluminescent immunoassay; ELISA: Enzyme-linked immunosorbent assay; RIA: Radioimmunoassay; EASIA: Enzyme Amplified Sensitivity Immunoassay.
