# Supplementary material for: Association between obesity and risk of fracture, bone mineral density and bone quality in adults: A systematic review and meta-analysis
Source: PLoS One. 2021 Jun 8;16(6):e0252487. doi: 10.1371/journal.pone.0252487 (PMC8186797; doi:10.1371/journal.pone.0252487)
Supplement: S4 Table — (DOCX) [file pone.0252487.s005.docx]

| **S4 Table.** Results of subgroup analysis by obesity and risk of bias criterion for bone mineral density and bone turnover markers outcomes in postmenopausal women, premenopausal women and men. | | | | | |
| --- | --- | --- | --- | --- | --- |
| **Outcome** | **Subgroup analysis** | **Number of studies** | **Effect estimate (95% CI)** | **Intra-groups I^2^ (%)** | **Subgroup difference I^2^ (%)** |
| **Postmenopausal women** | | |  |  |  |
| Any fracture | Obesity criterion | | | | |
|  | Overweight and obese  Obese only | 10  2 | 0.85 (0.73, 0.97)  0.93 (0.87, 1.00) | 97%  0% | 36.6% |
|  | Risk of bias | | | | |
|  | Low  Moderate  High | 7  5  0 | 0.80 (0.69, 0.92)  0.99 (0.85, 1.15)  - | 95%  86%  - | 77.1% |
| Hip fracture | Obesity criterion | | | | |
|  | Overweight and obese  Obese only | 6  2 | 0.71 (0.57, 0.89)  0.90 (0.78, 1.05) | 96%  24% | 66.5% |
|  | Risk of bias | | | | |
|  | Low  Moderate  High | 6  2  0 | 0.75 (0.61, 0.92)  0.78 (0.60, 1.02)  - | 96%  0%  - | 0% |
| Total hip aBMD | Obesity criterion | | | | |
|  | Overweight and obese  Obese only | 8  3 | 0.11 (0.08, 0.15)  0.10 (0.06, 0.13) | 97%  61% | 0% |
|  | Risk of bias | | | | |
|  | Low  Moderate  High | 6  6  - | 0.10 (0.08, 0.12)  0.12 (0.07, 0.16)  - | 58%  98%  - | 0% |
| Femoral neck aBMD | Obesity criterion | | | | |
|  | Overweight and obese  Obese only | 17  3 | 0.07 (0.05, 0.08)  0.05 (-0.04, 0.13) | 87%  96% | 0% |
|  | Risk of bias | | | | |
|  | Low  Moderate  High | 7  14  0 | 0.03 (-0.02, 0.07)  0.06 (0.05, 0.08)  - | 93%  91%  NA | 51.1% |
| Lumbar spine aBMD | Obesity criterion | | | | |
|  | Overweight and obese  Obese only | 17  8 | 0.07 (0.05, 0.09)  0.06 (-0.03, 0.15) | 88%  96% | 0% |
|  | Risk of bias | | | | |
|  | Low  Moderate  High | 8  18  1 | 0.04 (-0.02, 0.10)  0.08 (0.06, 0.10)  0.03 (-0.01, 0.06) | 94%  91%  NA | 71.9% |
| Radius aBMD | Obesity criterion | | | | |
|  | Overweight and obese  Obese only | 3  3 | 0.07 (0.04, 0.10)  0.06 (0.03, 0.09) | 55%  80% | 0% |
|  | Risk of bias | | | | |
|  | Low  Moderate  High | 3  3  0 | 0.06 (0.04, 0.08)  0.08 (0.05, 0.11)  - | 65%  60%  NA | 0% |
| Osteocalcin level | Obesity criterion | | | | |
|  | Overweight and obese  Obese only | 9  5 | -0.42 (-2.00, 1.17)  -2.33 (-4.70, 0.03) | 99%  65% | 42.7% |
|  | Risk of bias | | | | |
|  | Low  Moderate  High | 6  10  1 | -0.34 (-0.84, 0.16)  -1.40 (-3.38, 0.59)  -0.90 (-1.68, -0.11) | 57%  98%  NA | 5.8% |
| P1NP level | Obesity criterion | | | | |
|  | Overweight and obese  Obese only | 6  2 | -3.79 (-8.46, 0.88)  -2.44 (-9.33, 4.46) | 73%  0% | 0% |
|  | Risk of bias | | | | |
|  | Low  Moderate  High | 4  4  0 | -5.55 (-9.88, -1.21)  -2.59 (-8.17, 2.99)  - | 0%  78%  NA | 0% |
| CTX level | Obesity criterion | | | | |
|  | Overweight and obese  Obese only | 9  2 | -0.11 (-0.15, -0.06)  -0.01 (-0.18, 0.16) | 80%  42% | 9.7% |
|  | Risk of bias | | | | |
|  | Low  Moderate  High | 5  7  0 | -0.09 (-0.13, -0.04)  -0.09 (-0.14, -0.03)  - | 25%  82%  NA | 0% |
| **Premenopausal women** | | | | | |
| Total hip aBMD | Obesity criterion | | | | |
|  | Overweight and obese  Obese only | 6  3 | 0.06 (0.04, 0.08)  0.14 (0.11, 0.17) | 93%  0% | 94.3% |
|  | Risk of bias | | | | |
|  | Low  Moderate  High | 4  4  1 | 0.13 (0.10, 0.15)  0.06 (0.03, 0.08)  0.05 (-0.04, 0.13) | 15%  96%  NA | 86.9% |
| Femoral neck aBMD | Obesity criterion | | | | |
|  | Overweight and obese  Obese only | 11  2 | 0.05 (0.03, 0.07)  0.09 (0.04, 0.14) | 93%  24% | 58.5% |
|  | Risk of bias | | | | |
|  | Low  Moderate  High | 3  7  3 | 0.05 (0.03, 0.07)  0.04 (0.01, 0.07)  0.09 (0.07, 0.12) | 96%  88%  84% | 67.1% |
| Lumbar spine aBMD | Obesity criterion | | | | |
|  | Overweight and obese  Obese only | 12  5 | 0.06 (0.02, 0.09)  0.08 (0.05, 0.11) | 93%  0% | 5.4% |
|  | Risk of bias | | | | |
|  | Low  Moderate  High | 5  9  3 | 0.06 (-0.01, 0.12)  0.06 (0.03, 0.09)  0.08 (0.00, 0.17) | 88%  79%  73% | 0% |
| Radius aBMD | Obesity criterion | | | | |
|  | Overweight and obese  Obese only | 7  3 | 0.03 (0.01, 0.04)  0.03, 0.02, 0.04) | 89%  0% | 0% |
|  | Risk of bias | | | | |
|  | Low  Moderate  High | 4  3  3 | 0.04 (0.02, 0.06)  0.02 (0.00, 0.03)  0.02 (0.00, 0.05) | 80%  68%  86% | 29.7% |
| **Men** | | | | | |
| Any fracture | Obesity criterion | | | | |
|  | Overweight and obese  Obese only | 8  1 | 0.74 (0.61, 0.90)  1.00 (0.83, 1.20) | 90%  NA | 78.4% |
|  | Risk of bias | | | | |
|  | Low  Moderate  High | 5  4  0 | 0.76 (0.62, 0.93)  0.79 (0.51, 1.23)  - | 89%  93%  - | 0% |
| Hip fracture | Obesity criterion | | | | |
|  | Overweight and obese  Obese only | 5  1 | 0.52 (0.45, 0.61)  1.00 (0.83, 1.20) | 55%  NA | 96.3% |
|  | Risk of bias | | | | |
|  | Low  Moderate  High | 5  0  0 | 0.59 (0.44, 0.79)  -  - | 91%  NA  NA | NA |
| Total hip aBMD | Obesity criterion | | | | |
|  | Overweight and obese  Obese only | 7  2 | 0.07 (0.05, 0.08)  0.07 (0.05, 0.09) | 82%  78% | 0% |
|  | Risk of bias | | | | |
|  | Low  Moderate  High | 6  3  0 | 0.07 (0.06, 0.09)  0.05 (0.03, 0.06)  - | 77%  0%  NA | 88.6% |
| Femoral neck aBMD | Obesity criterion | | | | |
|  | Overweight and obese  Obese only | 7  1 | 0.05 (0.03, 0.08)  0.03 (0.00, 0.06) | 81%  NA | 42.0% |
|  | Risk of bias | | | | |
|  | Low  Moderate  High | 5  3  0 | 0.06 (0.03, 0.09)  0.03 (0.01, 0.05)  - | 85%  0%  NA | 71.8% |
| Lumbar spine aBMD | Obesity criterion | | | | |
|  | Overweight and obese  Obese only | 7  1 | 0.06 (0.04, 0.08)  0.04 (0.00, 0.09) | 55%  NA | 0% |
|  | Risk of bias | | | | |
|  | Low  Moderate  High | 5  3  0 | 0.06 (0.04, 0.08)  0.06 (0.02, 0.10)  - | 54%  56%  NA | 0% |

aBMD: areal bone mineral density; P1NP: Procollagen type 1 intact N-terminal propeptide; CTX: C-terminal telopeptide; CI: confidence interval.
