# Supplementary material for: Association between obesity and risk of fracture, bone mineral density and bone quality in adults: A systematic review and meta-analysis
Source: PLoS One. 2021 Jun 8;16(6):e0252487. doi: 10.1371/journal.pone.0252487 (PMC8186797; doi:10.1371/journal.pone.0252487)
Supplement: S1 Fig — Forest plot of pooled effect size for the risk of A) clinical vertebral fracture, B) wrist fracture, C) forearm fracture and D) ankle fracture in postmenopausal women with vs. without obesity, using a random-effect model. (DOCX) [file pone.0252487.s006.docx]

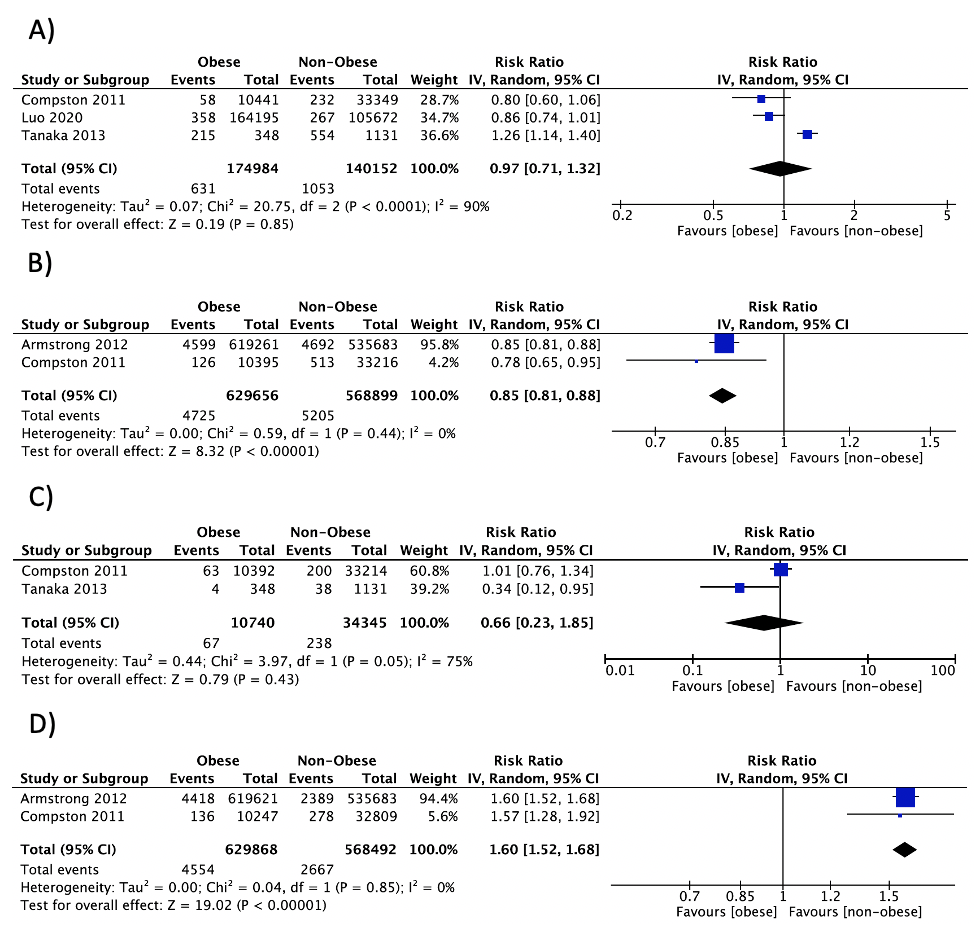


**S1 Fig**. Forest plot of pooled effect size for the risk of A) clinical vertebral fracture, B) wrist fracture, C) forearm fracture and D) ankle fracture in postmenopausal women with vs. without obesity, using a random-effect model.
