# Supplementary material for: Association between obesity and risk of fracture, bone mineral density and bone quality in adults: A systematic review and meta-analysis
Source: PLoS One. 2021 Jun 8;16(6):e0252487. doi: 10.1371/journal.pone.0252487 (PMC8186797; doi:10.1371/journal.pone.0252487)
Supplement: S3 Fig — Forest plot of pooled effect size for the lumbar spine aBMD by DXA mean difference between A) postmenopausal women, B) premenopausal women and C) men with vs. without obesity, using a random-effect model. (DOCX) [file pone.0252487.s008.docx]

**
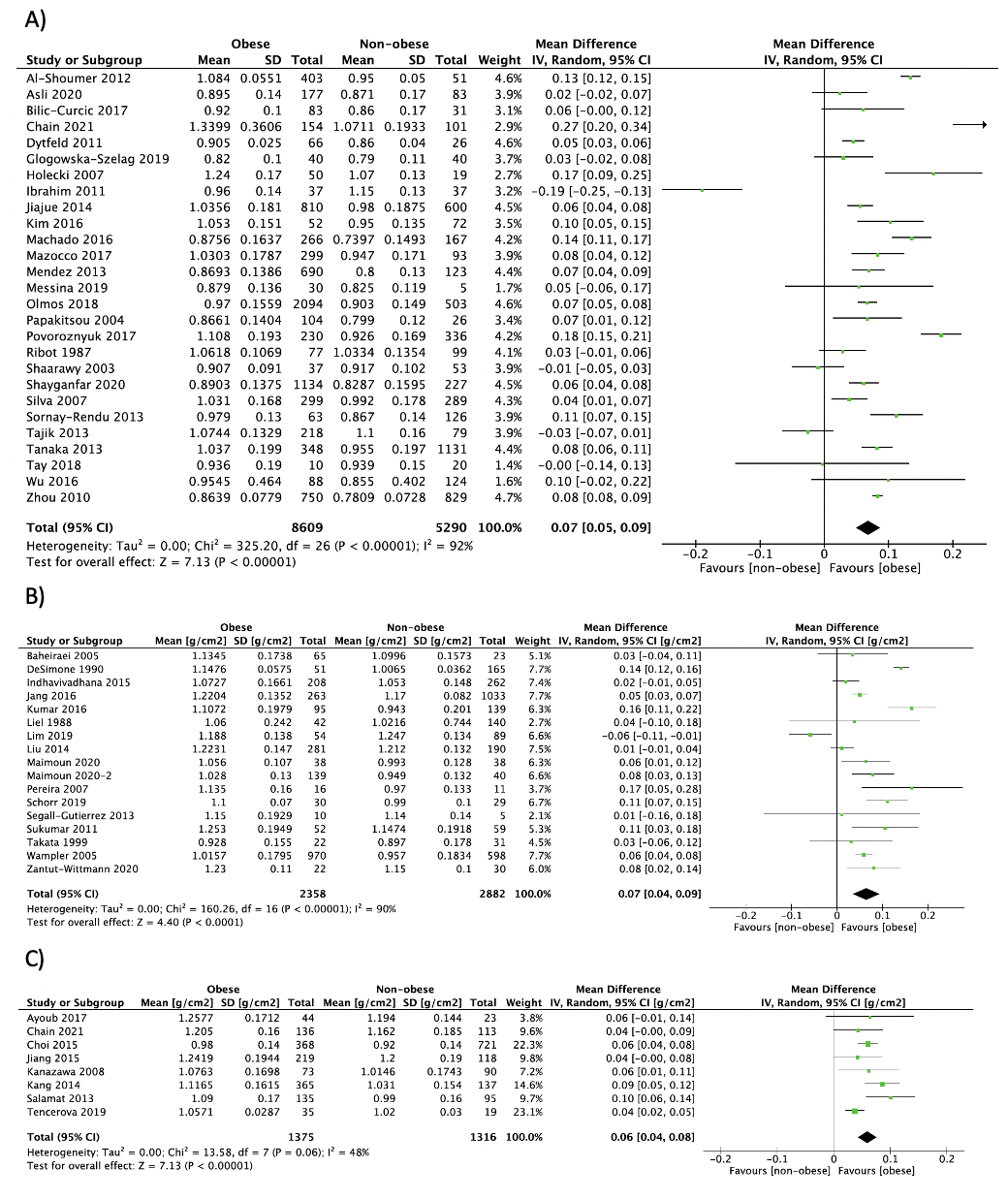
**

**S3 Fig**. Forest plot of pooled effect size for the lumbar spine aBMD by DXA mean difference between A) postmenopausal women, B) premenopausal women and C) men with vs. without obesity, using a random-effect model.
