# Supplementary material for: Association between obesity and risk of fracture, bone mineral density and bone quality in adults: A systematic review and meta-analysis
Source: PLoS One. 2021 Jun 8;16(6):e0252487. doi: 10.1371/journal.pone.0252487 (PMC8186797; doi:10.1371/journal.pone.0252487)
Supplement: S7 Fig — Forest plot of pooled effect size for A) P1NP levels mean difference between postmenopausal women with vs. without obesity, and total osteocalcin levels mean difference between B) postmenopausal women, C) premenopausal women and D) men with vs. without obesity, using a random-effect model. (DOCX) [file pone.0252487.s012.docx]

**
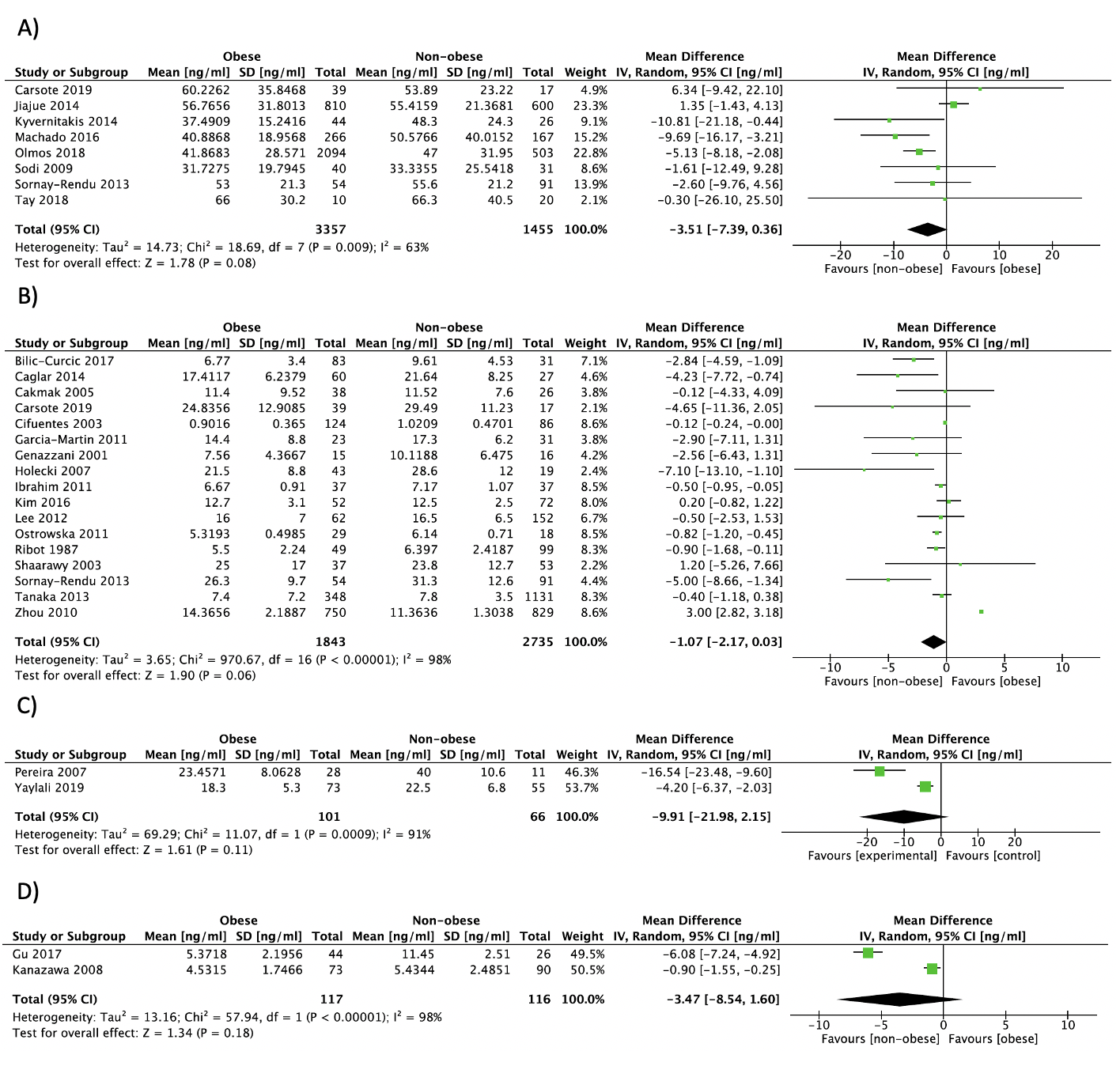
**

**S7 Fig**. Forest plot of pooled effect size for A) P1NP levels mean difference between postmenopausal women with vs. without obesity, and total osteocalcin levels mean difference between B) postmenopausal women, C) premenopausal women and D) men with vs. without obesity, using a random-effect model.
