# Supplementary material for: Association between obesity and risk of fracture, bone mineral density and bone quality in adults: A systematic review and meta-analysis
Source: PLoS One. 2021 Jun 8;16(6):e0252487. doi: 10.1371/journal.pone.0252487 (PMC8186797; doi:10.1371/journal.pone.0252487)
Supplement: S8 Fig — Forest plot of pooled effect size for A) CTX levels and B) NTX levels mean difference between postmenopausal women with vs. without obesity, using a random-effect model. (DOCX) [file pone.0252487.s013.docx]

**
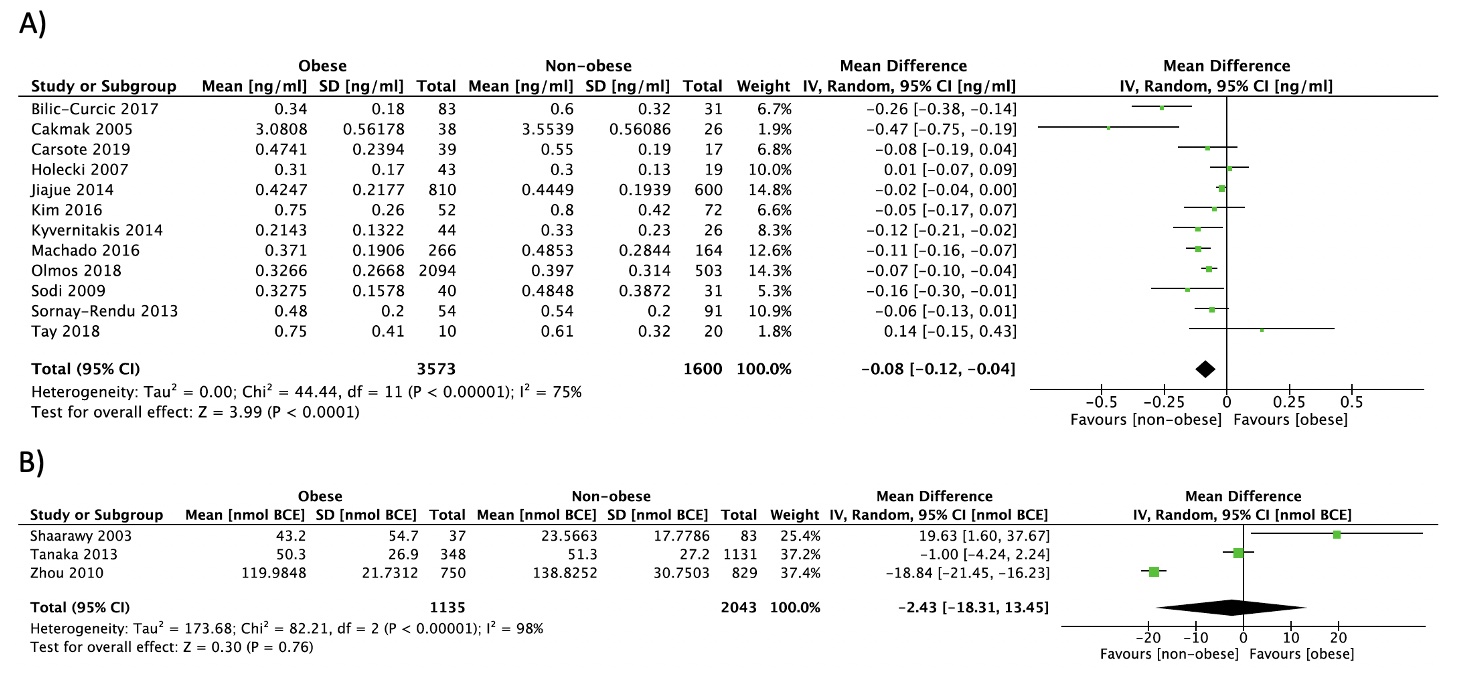
**

**S8 Fig**. Forest plot of pooled effect size for A) CTX levels and B) NTX levels mean difference between postmenopausal women with vs. without obesity, using a random-effect model.
