# Supplementary material for: Association between obesity and risk of fracture, bone mineral density and bone quality in adults: A systematic review and meta-analysis
Source: PLoS One. 2021 Jun 8;16(6):e0252487. doi: 10.1371/journal.pone.0252487 (PMC8186797; doi:10.1371/journal.pone.0252487)
Supplement: S13 Fig — (DOCX) [file pone.0252487.s018.docx]

**S13 Fig**. Funnel plot for femoral neck aBMD in postmenopausal women.
